# Bionic for Training: Smart Framework Design for Multisensor Mechatronic Platform Validation

**DOI:** 10.3390/s22010249

**Published:** 2021-12-30

**Authors:** Ruben Foresti, Rosario Statello, Nicola Delmonte, Francesco Paolo Lo Muzio, Giacomo Rozzi, Michele Miragoli, Leopoldo Sarli, Gianluigi Ferrari, Claudio Macaluso, Marcello Giuseppe Maggio, Francesco Pisani, Cosimo Costantino

**Affiliations:** 1 Department of Medicine and Surgery, University of Parma, 43126 Parma, Italy; ruben.foresti@unipr.it (R.F.); rosarioignazio.statello@studenti.unipr.it (R.S.); francescopaolo.lomuzio@univr.it (F.P.L.M.); giacomo.rozzi@jemtech.it (G.R.); michele.miragoli@unipr.it (M.M.); leopoldo.sarli@unipr.it (L.S.); claudio.macaluso@unipr.it (C.M.); marcellogiuseppe.maggio@unipr.it (M.G.M.); francesco.pisani@unipr.it (F.P.); 2 Department of Medical Sciences and Public Health, University of Cagliari, 09124 Cagliari, Italy; 3 Department of Engineering and Architecture, University of Parma, 43125 Parma, Italy; nicola.delmonte@unipr.it (N.D.); gianluigi.ferrari@unipr.it (G.F.); 4 Department of Surgery, Dentistry, Pediatrics and Gynecology, University of Verona, 37126 Verona, Italy; 5 Department of Molecular Cardiology, Humanitas Research Hospital, IRCCS, 20089 Rozzano, Italy

**Keywords:** bionic, neonatal apnea, three-axis accelerometer, zero-failure, 8Ws

## Abstract

Home monitoring supports the continuous improvement of the therapy by sharing data with healthcare professionals. It is required when life-threatening events can still occur after hospital discharge such as neonatal apnea. However, multiple sources of external noise could affect data quality and/or increase the misdetection rate. In this study, we developed a mechatronic platform for sensor characterizations and a framework to manage data in the context of neonatal apnea. The platform can simulate the movement of the abdomen in different plausible newborn positions by merging data acquired simultaneously from three-axis accelerometers and infrared sensors. We simulated nine apnea conditions combining three different linear displacements and body postures in the presence of self-generated external noise, showing how it is possible to reduce errors near to zero in phenomena detection. Finally, the development of a smart 8Ws-based software and a customizable mobile application were proposed to facilitate data management and interpretation, classifying the alerts to guarantee the correct information sharing without specialized skills.

## 1. Introduction

Recent studies showed that the healthcare system should adopt new innovative technologies [1] for patients’ remote assistance and monitoring [2], acquiring data in real time to design a predictive algorithm for future implementation of artificial intelligence [3,4,5]. The development of these tools [6,7] has to consider the zero-failure approach [8] in managing any information and related data mining to assure pathological phenomena detection [9] and reduction of errors over time. Thus, it is mandatory to schedule data-implementing semantic ontologies [10] identifying a continuous improvement and teaching process applicable to human and machine, in line with the Global Standard Method for Society 5.0 [8]. 

This model is difficult to adopt in remote conditions where both technologies and controlled environment (i.e., humidity, temperature, vibrations, etc.) are not certified for its specific use [11,12,13]. In this context, the autonomous region of interest identification of the specific phenomena require filtered data by clinicians. Therefore, there is the need to develop devices, with dedicated human–machine interface, able to avoid errors in detection and analysis without expensive sensors [14]. These sensors have to be downgraded in terms of risk level via feedback systems and smart algorithms capable of managing uncertain data [15]. Moreover, these smart devices have to improve skills [16] via educational tools such as video tutorials. 

To implement this type of self-assisted approach, we have to identify a real pathological case designing the related human-cyber-physical space [8] and the optimal management of processed data, simulating physical conditions via mechatronic devices. Finally, the obtained data have to be scheduled considering the 8Ws [8] to improve the quality and performance of the smart software. 

In this study, we focused on neonatal apnea, which is one of the most common cardiorespiratory events detected in newborns. Neonatal apnea is defined as a respiratory pause that lasts for at least 20 s or more than 10 s when coupled with low hemoglobin saturation and bradycardia [17,18]. Neonatal apnea can be caused by different underlying conditions (e.g., cardiac disease, infections, intracranial injury, metabolic disorders, and upper airway anomalies), but it often reflects the immaturity of the respiratory neural control. On the basis of the presence of brainstem-driven breathing efforts and airway obstructions, apneic episodes are traditionally classified as follows: (i) central apneas, (ii) obstructive apneas, and (iii) mixed apneas [13]. Although neonatal apnea may resolve within 40 weeks postmenstrual age, persistent episodes can still occur, prolonging hospital stay beyond this time, with monitoring at home potentially required [19]. Of note, delayed resolution of recurrent apnea might lead to worse neurodevelopmental outcomes [20], although life-threatening apneic events become rare after 43 weeks postconceptional age [21].

In the neonatal intensive care unit (NICU), vital signs of newborns at risk are continuously monitored through multi-sensor systems based on electroencephalography and polysomnography, and many different devices are commercially available for home monitoring [22]. Although several techniques (e.g., transthoracic impedance pneumography, pulse oximetry, respiratory inductance plethysmography, nasal thermistry, abdominal pressure tracing) can be implemented to detect neonatal apnea events, the majority sense artifacts and are relatively unable to detect obstructive apneas [13,23], increasing the risk of delay between phenomena detection and alert activation. Therefore, the availability of low-cost and easy-to-use monitoring devices together with parent-friendly education resources may be pivotal after hospital discharge.

In our work, we developed a mechatronic platform to simulate the movement (linear displacement and acceleration) of the abdomen during breathing to test the performance of sensors used to evaluate the presence or the absence of the movement of the abdomen occurring in non-obstructive apnea events. Therefore, we studied the spectrum of three-axis accelerometers and infrared sensors (IR) before and after introducing external noise with a servo-controlled hammer. Moreover, we reproduced the effect of soft materials (clothing) using additive manufacturing technology. 

We also applied the zero-failure approach [8] in the data acquisition to prevent any possible misdetection related to sensor reliability, which defines the answers to the 8Ws. 

Finally, we developed an Android application to educate the parents through informative tutorials and medical questionnaires without dedicated training, reducing at the same time the human–machine interaction gap [24]. 

In conclusion, this work aimed to propose a framework to test and measure sensors to capture non-obstructive apnea events. This approach could be applied to different clinical conditions and implemented in dedicated simulation-laboratories within innovative education program [25,26] of Hospital 4.0 [27].

## 2. Materials and Methods

### 2.1. Mechatronic Platform

**Figure 1:**
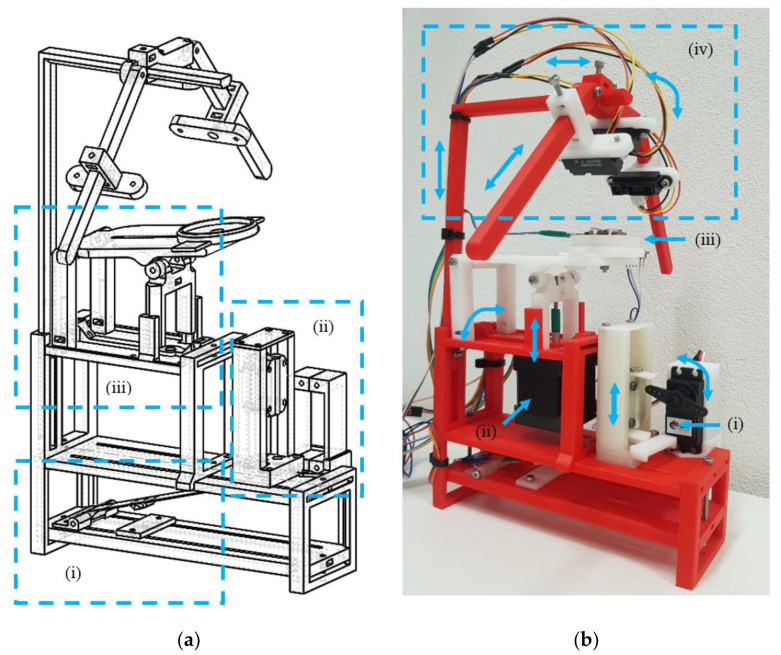
Mechatronic device for the physical simulation of the abdomen displacement: (**a**) schematics of the mechatronic platform, (**i**) vibration noise detector module, (**ii**) hammer module with adjustable energy, (**iii**) human motion/position simulator module; (**b**) 3D printed mechatronic platform, (**i**) servomotor, (**ii**) stepper motor, (**iii**) soft printed composite disc for the simulation of the physical response of clothes, (**iv**) infrared sensor support pole.

We realized a mechatronic platform composed of 3 sections: the first (Figure 1a(i)) is dedicated to the vibration noise detection, where a first accelerometer is placed directly in contact with the 3D printed ceiling. The second section (Figure 1a(ii)) generates disturbing vibrations using a hammer of known mass moved by a servomotor (Figure 1b(i)) with a continuous rotation DS04-NFC (torque: 5.5 kg/cm), enabling change of the speed, displacement, and energy transmitted to the mechatronic platform. The last section can rotate up to 180° (Figure 1a(iii)), reproducing the real positions of a newborn lying down on the bed and simulating the movement of the abdomen during breathing (Figure 1b(ii); linear displacement of 0.5–1.5; resolution 0.11µm/step; speed 0–120 bpm) via a hybrid stepper motor (nema 17, 17HS19-2004S1, torque: 5.9 kg/cm), controlled by the chopper stepper driver A4988 (Allegro MicroSystems, Worcester, MA, USA).

In this section, we also added soft 3D printed composite [28] discs (Figure 1b(iii)) to simulate the physical response of clothing or mattress. Soft components were developed via Freeformer 200-3X (Arburg, Loßburg, Germany) on the basis of db-PED technology by using Cawiton PR13630 medical-grade material (Rubberfabriek Wittenburg BV, Zeewolde, the Netherlands) [29] at extrusion/environmental temperatures of 210 °C and 50 °C, respectively, and printing speeds from 20 mm/s to 65 mm/s.

Finally, a support pole (Figure 1b(iv)) was designed for IR that does not require light and contact during detection.

All the mechanical parts were designed with SolidWorks^®^ v. 2015 (Solidsolution, London, UK). The mechanical components (red and transparent colors, Figure 1b) were fabricated using a custom-made 3D printer based on fused deposition modeling (FDM) technology (Bio-4esti) [30]. A dedicated extruder for bobbin filament with a 1.75 mm diameter was used to create the hard parts. The 3D model was printed using polylactic acid (PLA, 175N1, Velleman Inc., Legen Heirweg, Gavere, Belgium) extruded at 205 °C and on a printing platform at 60 °C. The printing speeds were set between 25 and 80 mm/s for external perimeters and filling, respectively. White 3D printed parts were fabricated via stereolithography (SLA) 3D printing technology (Formlabs2, Somerville, MA, USA) using rigid resin material (RIGID, Somerville, MA, USA). After the printing phase, resin objects were washed with isopropyl alcohol (GIP103, Girelli Alcool, Milano, IT) for 5 min and cured under UV light (HybriLinker, UVP, Upland, CA, USA) for 15 min.

### 2.2. Sensors, Microcontroller, and Android App

The accelerometers were chosen considering three-axis low and ultralow power Analog Devices’ products (Table 1), selecting the accelerometer printed circuit board (PCB) based on the ADXL335 (SparkFun, Electronics, Colorado, USA) integrated circuit because of its measurement range (±3 g) and sensitivity (682 LSB/g).

**Figure 2:**
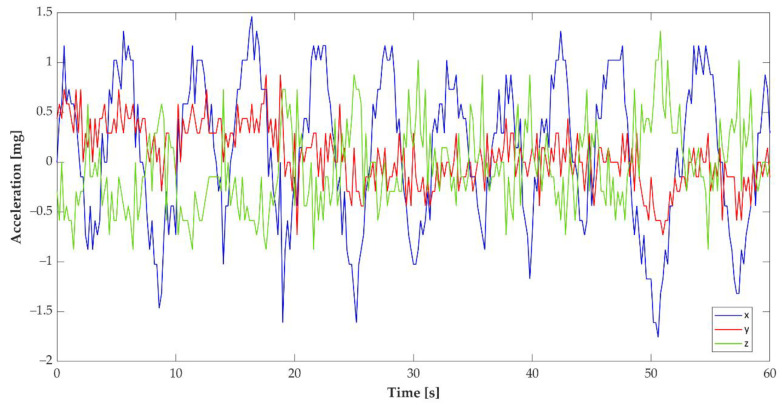
Accelerations measured along the 3-axis of an ADXL 335 worn by a volunteer (sampling rate = 5 samples/s) lying on a bed in the absence of external vibrations using a personal computer with a dedicated signal detector. In the example portrayed, the gravity acceleration was subtracted from the signal amplitude.

Figure 2 shows the signal obtained by placing the devices on the abdomen of a volunteer (among the authors of the work) to acquire the signals for the preliminary evaluation of the abdomen’s movement and its possible attenuations/noises/interferences evaluations. Moreover, the multisensory platform was equipped with the Sharp GP2Y0A41SK0F Analog Distance IR Sensor (Sharp Electronics, Osaka, Japan). Temperature and relative humidity can be measured using one DHT11 (20–90% RH, 0–50 °C) sensor. To manage the motion devices, we adopted an Arduino Mega 2560 Board (Arduino, Monza, IT) connected in parallel communication with an Arduino DUE Board that reads and saves the sensor values on the 16GB SD card (Kingston Technology, Fountain Valley, CA, USA). Finally, for the local communication with the human interface, we installed an HC-06 Bluetooth antenna (Guangzhou HC information technology Co., Guangzhou, China).

All the microcontrollers were programmed with Arduino SDK 1.8.5, and the GUI was developed using the MIT App Inventor 2 platform and installed on an Android tablet (Galaxy Tab A SM-T585, Samsung, Seoul, South Korea) equipped with 7.0 Android software version.

### 2.3. Statistical Analysis

The data were analyzed via Excel (Office365) and reported as mean ± SD. In detail, the χ^2^ test was performed for testing the relationship between categorical variables (i.e., “IR adoption” and “detection”). Moreover, we calculated the odds of detecting an error (unusable data/detected breathing) for each tested condition (i.e., for each different angle and displacement with or without IR sensor). Finally, the average value of the odds was calculated for each displacement (500 µm, 1000 µm, and 1500 µm) and for each angle (45°, 90°, and 135°). Statistical significance was set at *p* ≤ 0.05.

## 3. Results

We obtained a rapid prototyping tool and easy-to-scale platform, selecting two microcontroller boards for the management and analysis of events during monitoring (HCP Space). The PCBs operate in parallel, one is equipped with an ATMega 2560 while the second with an ATSAM3X8E.

**Figure 3:**
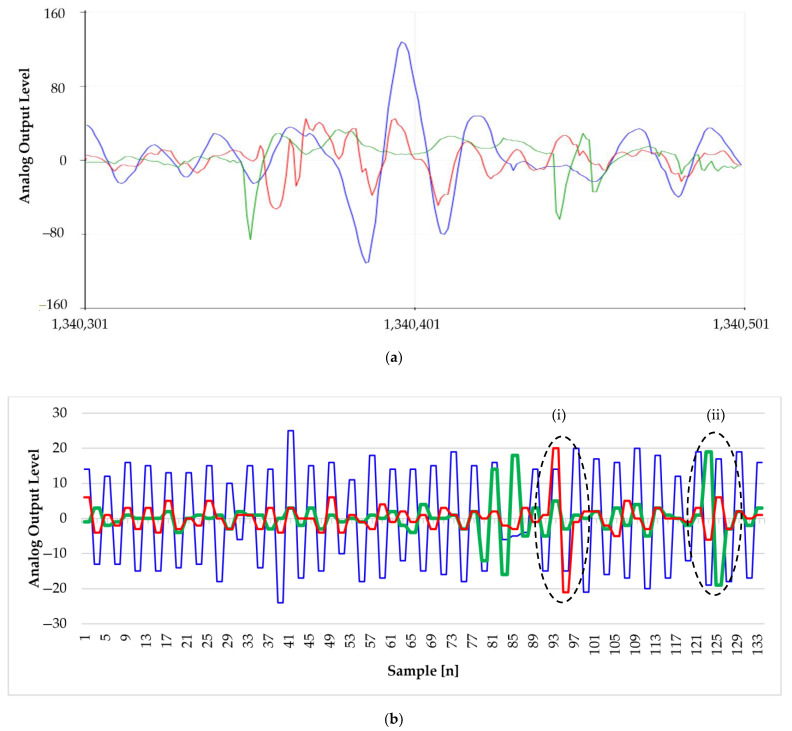
Representative signals acquired via multi-sensor accelerometers. (**a**) Signals plotted by Arduino SDK serial plotter. (**b**) Simulation of external noise generated via the hammer module by changing the displacement ((i) and (ii), respectively). Data were stored on a SD card and plotted with Microsoft Excel software. For both graphs, the *x*-axis is in number of sample and the *y*-axis represents the amplitude in bit. Green line: ceiling noise; red line: bed noise; blue line: signal detected by the accelerometer fixed on the module emulating the abdomen movement.

Adopting the cheaper ADXL 335 (Table 1) and exploiting the 12 bits ADC of an ATSAM3X8E-based board, we were able to obtain a sensitivity of 682 LSB/g with a scale factor of around 1.46 mg/LSB. This sensor, coupled with the two microcontrollers, represents a good trade-off between costs and performances, assuring the design lower limit in terms of resolution and sampling. In this way, the software could be easily scaled on a multicore and high-resolution platform (i.e., ESP 32), but a custom PCB will be needed to manage the high power required for engine motion control. Moreover, the proposed platform is already designed to be used with bionic device, such as a 3D printed newborn, by inserting the system inside it. The signal of the movement of the abdomen during sleep simulation was easily identified (Figure 2 and Figure 3a,b) through the ADXL 335, assuring the absence of external vibrations.

Using the aforementioned platform, we were able to simultaneously acquire three analog inputs (Figure 3a), maintaining a sampling frequency over 26 kHz. In this way, it was possible to evaluate the noise on the *z*-axes from each accelerometer. Therefore, we reproduced external disturbances to support the selection of the shock absorber able to damp it. In detail, Figure 3b represents the movement of the abdomen during breathing in the presence of noise generated through the servo-assisted hammer, highlighted by the superimposition of the two signals. In this test, the three accelerometers were placed in parallel to the support floor simulating a newborn in the supine position and considering his *z*-axis. 

To analyze any possible position on the bed, we obtained a final sampling frequency over 4kHz, acquiring simultaneously six analogue signals from our smart controller: all three coordinates from the abdomen accelerometer, only the z-coordinate from the ceiling/bed accelerometers, and the related IR sensor located perpendicularly to the abdomen accelerometer. 

We also simulated the pause of the abdomen’s movement, occurring in neonatal apnea, by stopping the stepper motor for more than 10 s to classify and schedule the uncertain data when the noise was superimposed to the accelerometer signal. In detail, we tested nine different conditions: three human motion-position and three linear displacement values (Table 2). For each test, we simulated 100 breathing repetitions, assumed as linear movements, and three random “apnea events” defined as absence of motion for 20 s.

We classified as unusable data the episodes where the signal of the noise accelerometer was higher in amplitude than the one acquired from the simulation platform. 

The results suggested that apneas characterized by different abdominal displacement (i.e., 500 µm, 1000 µm, and 1500 µm) could be identified in any position (i.e., human position module of 45°, 90°, and 135° angles), reaching errors of 4.24 ± 3.11% and 11.02 ± 5.41% with and without the use of IR sensor, respectively. Moreover, a significant relationship between “detection” and “IR sensor adoption” was found (detected without-IR sensor: 812 vs. detected with-IR sensor: 864; unusable data without-IR sensor: 88 vs. unusable data with-IR sensor: 36; χ^2^ _(1, n = 1800)_ = 23.42; *p* < 0.001). Specifically, on the basis of the odds ratio, we found that the odds of detecting an unusable data was 2.60 times higher if the system was not equipped with an IR sensor.

We observed an inverse correlation between displacement (500 µm, 1000 µm, and 1500 µm) and uncertain data (16.76 ± 2.09%, 10.30 ± 0.70%, 6.02 ± 1.31%) that could be reduced (7.54 ± 1.16%, 3.82 ± 1.25%, 1.35 ± 0.60%) using the accelerometers simultaneously with the IR sensor. As expected, it was easier to identify the abdomen displacement in the inclined positions (45° and 135° angles) because of a multi-axis detection of the phenomena.

To avoid the EEPROM saturation, we added the card reader for the real-time data storing, enabling the main part of the software to only analyze and detect the phenomena. 

**Figure 4:**
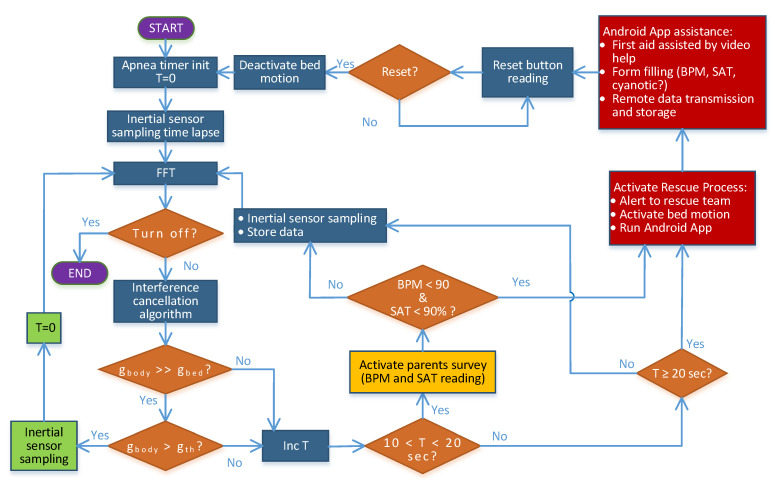
Proposed flowchart of the monitoring strategy to manage the data during an apnea event.

Moreover, we proposed the following scheme to manage data and apnea episodes (Figure 4).

At the beginning of the monitoring, a timer (T) is started to count the duration of delays between breaths. We simultaneously acquire the signals from accelerometers and IR (perpendicular to the abdomen accelerometer) to reduce uncertain data, as described in the simulation experiment. When the breathing activity cannot be identified due to interferences, the timer is never set to zero. If the acceleration measured by the accelerometer applied on the abdomen (g_body_) is greater than the one measured on the bed (g_bed_), it means that the signal from the breathing sensor is clearly detected. Then, it can be compared with a threshold level (g_th_), indicating the acceleration value related to displacement, in order to verify if there is an apnea or not. In the case of T being less than 10 s (which is the limit risk condition), the timer is reset and the monitoring loop starts again. If T is between 10 and 20 s, an apnea alert is generated, and the platform activates the parent survey to acquire both saturation (SAT) and beats per minute (BPM) to evaluate the real status of the newborn. When both SAT and BPM are below 90, we are in a critical condition and the rescue process will be activated. On the contrary, the movement analyses restart. 

When it is not possible to obtain a clear g_body_ signal for more than 20 s continuously, the timer is updated, activating the rescue process sending an alert to an Android app on the dedicated tablet. Then, the software enables the hardware port and its coupled relay to control any installed automatic actuator (bed motion). The mobile app counts the number of alerts (extracted from data uncertain or not), stores all the available data (temperature, humidity, acceleration, and displacement), and runs the user interface for the application to help a non-expert rescuer. 

**Figure 5:**
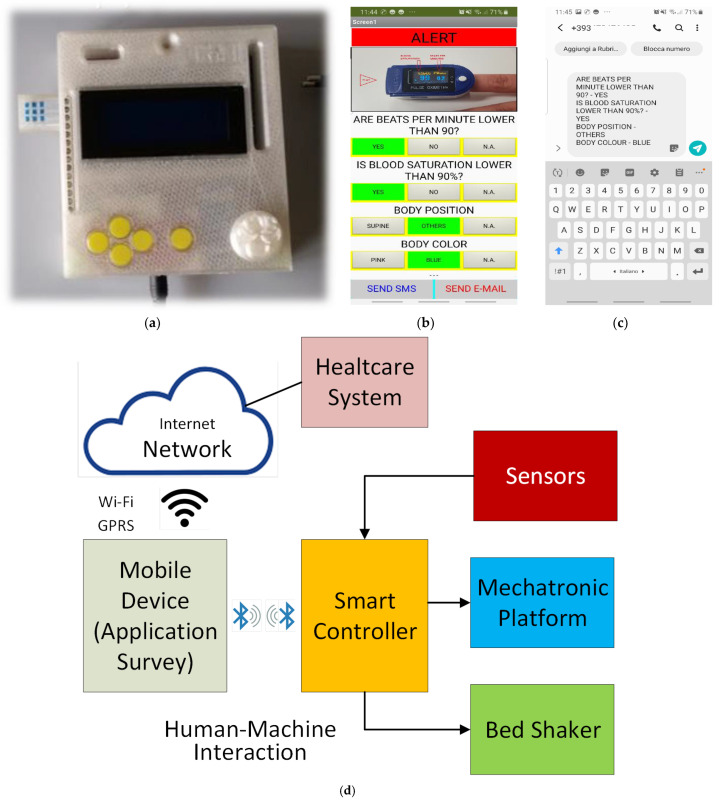
Smart platform: (**a**) smart controller, (**b**) mobile application survey, (**c**) SMS text automatically generated, (**d**) smart framework block diagram.

The smart controller (Figure 5a) was equipped with a human–machine interface connected via a Bluetooth module assuring a proximity control. In this way, we can prevent any possible intrusion and software corruption. Moreover, in case of a lost connection, the mobile app tries to reconnect itself, and if it fails, a sound alert is activated.

Following the above-mentioned data flow, the care provider receives the alert on the patient’s condition which generates a direct link to a video tutorial for the first intervention and a dedicated survey (Figure 5b). The simple form can be filled to record the events and other useful data such as the beats per minute, the blood saturation level, body positioning, and the patient’s skin color, and the data will be transmitted to a remote expert team. At the end of this process, the monitoring can be restarted by pressing the reset button.

The answers are classified via a risk color code (red: immediate intervention; yellow: planned check-up; green: prospective study), sending an automatic short message (Figure 5c,d) or e-mail to the healthcare system, improving the monitoring reliability over time and reducing the intervention delay.

Then, the data are stored directly on the parent’s and doctor’s tablets without the necessity of a server reducing the related costs. 

Finally, to reduce the variability of a possible predictive algorithm, the doctor can filter the information on the government’s cloud after a professional visit, overcoming the subjectivity of the survey’s answers.

In detail, the smart platform has to handle the 8Ws [8] collecting the smart data mined as described in Table 3. In this way, any smart software should be able to suggest the best strategy considering the acquired information (i.e., available tools, parameters), assuring the required continuous improvement. 

## 4. Conclusions

In clinical trials, ethical issues hamper the characterization of the sensors directly on patients. Therefore, we developed a mechatronic platform to simulate the absence or pause of the abdomen’s movement (breathing), which occurs in the non-obstructive apnea, in order to assure a reliable and standardized method to evaluate the effectiveness of multisensor systems. We designed a 3D printed structure with two degrees of freedom to reproduce the movement of the abdomen and body positioning. Moreover, we implemented soft printed elements to reproduce the physical effect of mattress and clothing. 

The designed platform included three-axial accelerometers MEMS and three IR sensors. One accelerometer detects the noise generated from the platform, the second enables the evaluation of different types of shock absorbers (to install at the base of the bed), and the last sensor analyzes the movement of the abdomen during breathing. Finally, the IR sensor, properly placed perpendicularly to the abdomen, supports the detection of the phenomena reducing the uncertain data in the presence of noise introduced by changing the parameters of the hammer module.

Moreover, the electronic PCB is equipped with humidity/temperature sensors, as well as relays for the activation of external devices, such as a bed shaker or the climate control of the room. Finally, to avoid any hacker attack, we adopted a Bluetooth antenna for the local communication with the tablet. 

The installed mobile application displays the survey, educates the untrained user with a video tutorial, and reduces the delay for the first aid via automatic message editing. The clinician can filter and store the acquired data and, when required, improve the information quality, customizing the survey. 

The proposed framework and bionic-based simulator could be scaled to a medical simulation laboratory using 3D printed morphological models extracted from patient CT-scan or MRI (digital bio-library) to simulate pathology and the related medical procedure. Finally, considering human habits during training, data are scheduled via 8Ws, and the mobile app could generate a quiz for the evaluation of the improved skills. 

## Figures and Tables

**Table 1:** Main features of the accelerometers by Analog Devices considered for the application proposed here.

Model	Type	Measurement Range	Output Resolution	Sensitivity	Scale Factor
ADXL 335	Analog	±3 g	-	300 mV/g ^1^	-
ADXL 345	Digital	±2 g	10 bit	256 LSB/g	3.9 mg/LSB
ADXL 350	Digital	±1 g	10 bit	512 LSB/g	1.95 mg/LSB
ADXL 313	Digital	±0.5 g	10 bit	1024 LSB/g	0.952 mg/LSB

^1^ With a voltage supply vs. = 3 V.

**Table 2:** Mechatronic breathing simulations: accelerometer detection versus multisensory detection.

Angle	Displacement (µm)	Detected Breathing (no IR)	Unusable Data (no IR)	Detected Breathing	Unusable Data
45°	500	86	14	93	7
	1000	91	9	97	3
	1500	93	7	99	1
90°	500	84	16	92	8
	1000	90	10	95	5
	1500	95	5	98	2
135°	500	87	13	94	6
	1000	91	9	97	3
	1500	95	5	99	1

**Table 3:** Healthcare 4.0—8Ws and related reply for neonatal apnea smart scheduling.

What Has Happened?	Where Is the Problem?	Why Has It Happened?	Who Can Restore It?	What to Do?	Which Devices and Tools to Use?	When to Do It?	How to Do It Well?
Colors	Smart platform geolocation	Phenomena discrimination	Available devices and resources	Operative and validated protocols	According to acquired data and available devices	Timing and related impact on delay and quality results	Big data analysis and continuous improvement

## Data Availability

Data will be provided upon request.
